# Disparities in Clinical Trial Enrollment– Focus on CAR-T and Bispecific Antibody Therapies

**DOI:** 10.1007/s11899-024-00747-6

**Published:** 2024-12-04

**Authors:** Nadia Islam, Laura Budvytyte, Nandita Khera, Talal Hilal

**Affiliations:** 1 Mayo Clinic Alix School of Medicine, Scottsdale, AZ 85254 USA; 2 Division of Hematology/Oncology, Mayo Clinic, 5777 E. Mayo Blvd, Phoenix, AZ 85054 USA

**Keywords:** Disparities, Clinical trials, Lymphoma, CAR-T, Bispecific antibody, Bispecifics, Diversity

## Abstract

**Purpose of Review:**

Recent studies show that unresolved disparities hinder enrollment to clinical trials, equitable distribution of treatments, and impact the generalizability of trials, compromising health outcomes across different populations. This review aims to examine the persistent disparities noted in clinical trial enrollment, with particular focus on lymphoid malignancies, CAR-T cell and bispecific antibody therapies.

**Recent Findings:**

Targeted interventions can enhance recruitment of underrepresented groups in clinical trials and address the complex barriers hindering participation, which are essential for achieving healthcare access equity and treatment outcomes.

**Summary:**

Improvement must be multifaceted, addressing socioeconomic, geographic, and biologic factors contributing to underrepresentation. This includes more lenient eligibility criteria, improving outreach and education, as well as using technology to diversify trial participation.

## Introduction

Clinical trials are integral to advancing medicine and introducing new therapies that are safe and effective. Results from these clinical trials often serve as the basis for decisions of drug approval by regulatory agencies such as the U. S Food and Drug and Administration (FDA). Following drug approval, the types of patients who are offered this intervention (e.g. drug or device) may be different than the types of patients enrolled in these pivotal clinical trials with regards to gender, race, comorbidities, or geographic distribution. These differences may or may not be significant enough to alter the expected efficacy of the intervention, but having a cohort of patients enrolled in trials that is at least somewhat representative of the real-world population ensures that results from these studies are generalizable to the overall population. Failure to achieve diversity in enrollment not only limits generalizability of results, but also exacerbates non-equitable access to novel therapies.

The past 30 years have seen legislative efforts aimed at increasing diversity in clinical trials. These efforts included the NIH Revitalization Act which passed in 1993, which established the Federal legislative mandate that NIH-funded research would be conducted such that variables studied in trials affect members of minority groups [1], the DEPICT Act which was a legislation proposed to increase transparency and accountability in clinical trials by reporting demographics and implementing proactive diversity action plans from clinical trial sponsor with engagement strategies to improve access to trials in underrepresented communities [2], and the NIH Clinical Trial Diversity Act, which is a more recent piece of legislature passed in May of 2023 aimed at establishing measurable goals for the recruitment and retention of participants presented [3]. Despite these initiatives, disparities persist, with the proportion of racial and ethnic minorities participating in trials persistently lower than the proportion of minorities in the US population at large (36.3%) [4]. Data on the disproportionate enrollment of patients of poor socioeconomic status (SES), those with comorbidities, or the elderly are lacking.

The US Food and Drug Administration (FDA) issued draft guidance in April 2022 calling for improvement of clinical trial enrollment of participants from minority racial and ethnic populations. This greater scrutiny is evident in the difference in reporting race and ethnicity in published trial reports. For example, the pivotal trial that led to the approval of chimeric antigen receptor T (CAR-T) cell therapy in the 3rd line setting in diffuse large B-cell lymphoma (DLBCL) [5–7] did not report information on race and ethnicity, but those that sought FDA approval of CF CAR-T cell therapy in the 2nd line setting of DLBCL do [8–10].

This review aims to evaluate literature on disparities in hematology clinical trials focusing on lymphoma and CAR-T/bispecific therapy, highlighting barriers to enrollment and proposing strategies for improvement. Because of limited information available in this area specifically for lymphoma, we do extrapolate data about these issues from other cancer types.

## Defining the Problem of Enrollment Disparities

**Fig. 1:**
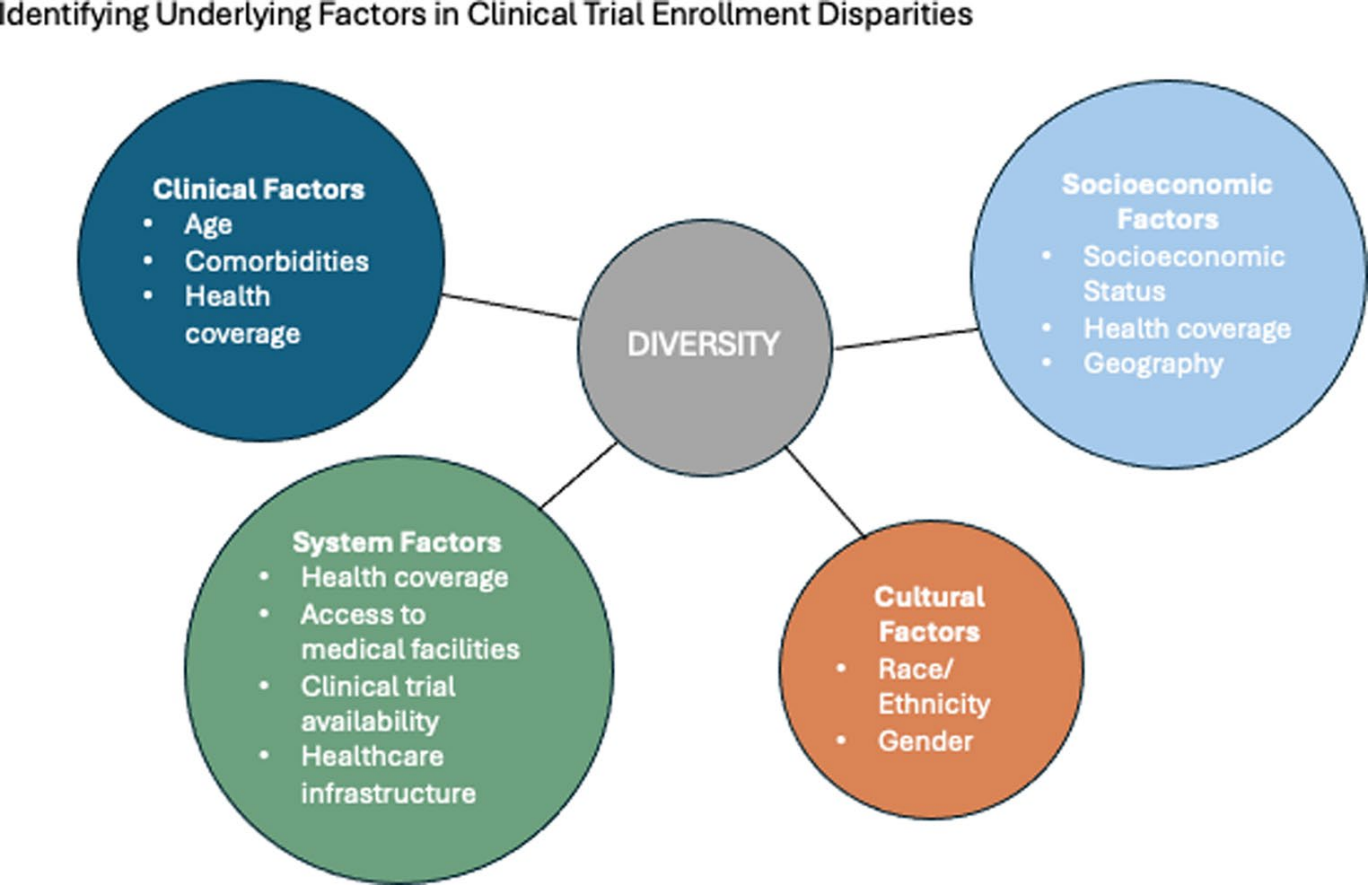
Categories of factors that contribute to disparities in clinical trial enrollment

Diversity is an overarching term which includes the demographic features of an individual and other differences between patient populations. Understanding the lack of diversity in lymphoma trials largely comes from research on other types of cancer. Numerous studies have investigated the issue of disparities in clinical trial enrollment, focusing on factors such as age, race/ethnicity, gender, comorbidities, SES, health coverage, and geography (Fig. 1, Table 1). The advent of high cost, medically complex treatments such as CAR-T cell therapy for lymphoma has highlighted the need to closely examine and address factors associated with disparities in access; clinical trial enrollment being one of the surrogates for access to quality cancer care.

**Table 1:** Disparities in race/ethnicity and socioeconomic factors in clinical trials of lymphoid malignancies and barriers to car-t/bispecific trial enrollment

Reference	Date	Type of Study	Aim of Study	Barriers	Key Findings
Faruqi et al. [16]	2022	Post-hoc analysis of prospective trials	To evaluate the impact of demographics and obesity on CAR T-cell therapy outcomes in adult and pediatric patients with hematologic malignancies treated with CAR T cell therapy across 5 phase 1 clinical trials at the NCI from 2012 to 2021	Race, ethnicity, obesity/body mass index	• Of 138 B-ALL CAR T-cell infusions, 28.8% of patients were Hispanic, and 3.6% were Black. • Hispanic patients had a higher likelihood of severe cytokine release syndrome compared to White non-Hispanics (odds ratio 4.5, *p* = 0.001). • In two landmark phase 3 US trials, 80% of patients were White, and less than 10% were Hispanic or Black. • Significant association was found between Hispanic ethnicity and severe cytokine release syndrome, independent of BMI.
Baggott et al. [45]	2021	Retrospective cohort	To explore outcomes of Black acute lymphoblastic leukemia patients treated with CAR T cell therapy	Race, ethnicity	• Of 185 B-ALL patients treated with CD19 CAR T-cell therapy, 5.9% were Black children and young adults. • Black patients were less likely to receive CAR T infusion and had worse outcomes compared to non-Black patients. • Black patients had more pre-CAR T relapses (median 2 vs. 1, *p* = 0.0105) and lines of therapy (median 5 vs. 2, *p* < 0.0001). • Higher rates of pre-CAR T SCT were observed in Black patients (71% vs. 24%, *p* = 0.0122). • Black patients were more likely to be infants at diagnosis (27% vs. 7%, *p* = 0.0468).
Kahn et al. [46]	2018	Prospective cohort	To compare treatment-related toxicities, event-free survival, and overall survival between Hispanic and non-Hispanic children with ALL	Race, ethnicity	• Hispanic children treated for ALL had fewer bone-related toxicities, but inferior survival compared to non-Hispanics. • Both biologic and non-biologic mechanisms affecting drug delivery and exposure may be important factors.
Ahmed et al. [17]	2022	Retrospective cohort	To determine patterns of racial/ethnic distribution, socioeconomic strata, insurance coverage and travel time of CAR T cell recipients	Race, ethnicity, socioeconomic status	• Blacks were less likely than other racial/ethnic groups to receive CAR T-cell therapy. • Blacks and Hispanics were underrepresented with 1% of CAR T recipients being Black, 5.4% Hispanic, and 7.3% from low-income neighborhoods. • About 1/3 of CAR T-cell recipients lived more than 2 h from their treatment center.
Flowers et al. [22]	2014	Retrospective population based	To examine the relationships between socioeconomic status, insurance status, demographic factors and diffuse large B-cell lymphoma (DLBCL) overall survival.	Socioeconomic factors, race. ethnicity	• Patients in lower SES neighborhoods had higher risks of all-cause and lymphoma-specific death compared to higher SES. • Uninsured and government-assisted patients had a 1.5x increased risk of death. • Fewer publicly insured individuals enrolled in CAR-T trials. • SES impacts mortality in curable diseases like DLBCL. • After adjusting for various factors, Hispanics, Asian/Pacific Islanders, and Blacks had higher all-cause mortality rates compared to non-Hispanic Whites.
Snyder et al. [23]	2021	Cross sectional study	To assess how expanding access to CAR- T cell therapy administration impacts patient travel distances and time	Geographic, socioeconomic	• Of 3922 CAR-T cell therapy candidates, > 37% traveled more than 1 h to the nearest academic hospital. • Expanding access to community hospitals and broader treatment centers reduced average travel time by 23% and distance by 30% (*p* < 0.001), with a 7% decrease in time and 8% decrease in distance (*p* < 0.01). • Expanding access could improve sociodemographic and rural-urban equity in CAR-T therapy allocation.
Flowers et al. [21]	2012	Retrospective cohort study	To analyze disparities in the diffusion of chemoimmunotherapy for DLBCL	Socioeconomic status, health insurance, facility characteristics, race, ethnicity	• Patients with localized disease, diagnosed in 2001, or who were Black, uninsured/Medicaid insured, or of lower SES were less likely to receive chemotherapy (all *P* < 0.0001). • Black patients, those over 60 years old, or with localized disease were less likely to receive chemoimmunotherapy (RR: 0.83, 0.94, 0.89, respectively).
Fiala et al. [47]	2017	Retrospective cohort study	To review the use of patterns of stem cell transplant and bortezomib using the Surveillance, Epidemiology and End Results (SEER)-Medicare linked database	Race, bias	• In multiple myeloma, treatment disparities aren’t fully explained by access barriers alone. • Blacks were 49% less likely than Whites to undergo stem cell transplant for MM and 37% less likely even after adjusting for income and insurance, suggesting other barriers like cultural factors or physician bias.
Zafar et al. [24]	2013	Prospective cohort study	To describe experiences of insured cancer patients requesting copayment assistance and describe the impact of health care expenses on well-being and treatment	Socioeconomic status	• Insured cancer patients seeking copayment assistance face significant financial burden. • 75% of insured CAR-T therapy patients applied for medication assistance and experienced lifestyle changes, • 68% cut back on leisure activities • 46% reduced spending on food and clothing • 46% used savings to cover out-of-pocket expenses • 20% took fewer prescribed medications
Snyder et al. [25]	2021	Cross-sectional study	To estimate travel-related economic burden associated with options among patients with relapsed/refractory DLBCL	Socioeconomic status, race, ethnicity	• Of 3922 CAR-T therapy candidates, over 37% traveled more than 1 h to the nearest academic hospital. • Expanding access to community hospitals reduced travel time by 23% and distance by 30% (*p* < 0.001), with a 7% decrease in time and 8% decrease in distance (*p* < 0.01). • Expanding access could improve sociodemographic and rural-urban equity in CAR-T therapy allocation.

### Age

Age disparities in clinical trials challenge the generalizability of research findings, as older adults are often underrepresented in studies despite being the largest consumers of healthcare services [11]. Exclusion of elderly individuals from trial enrollment is due to various factors including stringent eligibility criteria, functional impairments and comorbidities, systemic factors such as lack of resources and funding, physician factors, and patient factors [12]. This lack of representation can lead to extrapolation of efficacy and safety of treatments in older populations, resulting in less effective treatment outcomes for this population [12]. To effectively include older adults in cancer clinical trials, strategies can include designing trials specifically for this population/ broadening eligibility criteria, modifying designs for better data collection, conducting cohort studies, enhancing post-marketing surveillance, evaluating biological age, running differential dosing trials,, and advancing regulatory and policy efforts [12]. Addressing these disparities is crucial to ensure that clinical guidelines and medical interventions are applicable to all age groups.

### Race and Ethnicity

In trials sponsored by the National Cancer Institute evaluating a variety of cancers including breast, colorectal, lung, and prostate between 2000 and 2022, lower enrollment fractions were noted in Hispanics (odds ratio [OR] vs. whites of 0.72; 95% CI 0.68–0.77) and black individuals (OR 0.71; 95% 0.68–0.74) [1]. Between 1996 and 2002, the proportion of trial participants who were not white declined. A similar pattern was seen from a more modern nationwide cohort between 2017 and 2022 from approximately 800 sites of care from US cancer clinics [13]. The problem becomes more complex when assessing individual cancers. For example, Black individuals have lower enrollment in breast, lung, and colorectal cancer clinical trials, but similar enrollment in prostate cancer trials when compared to whites, indicating that equitable participation is achievable. The recruitment methods used for prostate cancer trials could be helpful in addressing the disparities seen in trials for other types of cancer [1]. When examining disparities among patients with hematologic cancers, Hispanic and Black individuals were 40-50% less likely to participate in clinical trials compared to whites [14]. A review of clinical trials conducted by Blood and Marrow Transplant Clinical Trials Network (BMTCTN) from 2014 to 2021 showed that the major barrier from underrepresented groups to enroll was being able to access HCT as a therapy [15]. Patients from underrepresented groups were less likely to be enrolled in BMT trials than other groups across all trials except the trial for haploidentical transplants where the proportion of enrolled minority patient was substantial [15].

Lack of representation is similarly evident in trials of CAR-T cell therapy for lymphoma, myeloma and acute lymphoblastic leukemia. Using the Vizient Clinical Database to examine pattern of racial/ethnic distribution of CAR-T cell therapy recipients, Black individuals were less likely than any other racial or ethnic group to receive CAR-T cell therapy, composing only 1% of the patients enrolled on multiple myeloma CAR-T trials vs. 16.6% in the non-CAR-T group [16, 17]. This underrepresentation is particularly notable in landmark phase 3 trials of CAR T-cell therapy in the United States, where a significant majority of participants are white, with Hispanic and Black representation below 10% [8–10]. Given the potential benefits of CAR T-cell therapy for chemotherapy-resistant high-risk patients, accessibility for individuals of diverse ethnic backgrounds is needed.

### Gender

Women have historically faced underrepresentation and undertreatment in medicine, particularly evident in clinical trials. A study examining nearly 20,000 clinical trials conducted between 2000 and 2020 found that women were enrolled in fewer oncology trials compared to their actual burden of disease [43]. Despite comprising 46.5% of the burden of disease, women accounted for only 42.9% of clinical trial participants [44]. This disparity raises concerns about the generalizability of trial results and the effectiveness of treatments for women [44].

Furthermore, funding allocation in trial research tends to favor men over women in a significant majority of cases. Approximately 75% of the time, funding is skewed towards research involving male participants with men overrepresented in cost allocation [44]. This funding bias perpetuates the underrepresentation of women in clinical research, limiting opportunities to understand gender-specific responses to treatments and potentially leading to suboptimal healthcare outcomes for women.

### Comorbidities

Having one or more comorbidities also contributes to decreased clinical trial participation as trials set inclusion/ exclusion criteria which prohibit those individuals from enrolling. This is complicated by a lack of empirically based standards for participant selection [18]. For example, one single-institution study of 235 Black American cancer patients found that only 20 patients (8.5%) being eligible based on inclusion criteria; most were excluded for co-existing comorbidities [19]. Although some comorbidities are understandably prohibitive of receipt of clinical trial therapy some may be too restrictive.

Additionally, the influence of comorbidities on trial participation is an important consideration. In a study from the Health Information National Trends Survey SEER which included approximately 1000 patients, only about 15% reported trial discussion and 8% reported being enrolled. Having one or more comorbidities was significantly associated with lower trial discussion [20].

### Socioeconomic Status and Geography

Socioeconomic factors perhaps play the most significant role in widening disparities in trial enrollment [21–24]. Individuals from lower socioeconomic backgrounds face additional barriers to accessing clinical trials, including financial constraints, lack of insurance coverage, and geographic or logistical challenges [17, 22–26]. In trials of hematologic malignancies, the racial and ethnic disparities are often attributed to social factors (e.g. community practice, vehicle ownership, limited English proficiency [14]. Compared to patients living in higher SES neighborhoods, patients with diffuse large B-cell lymphoma (DLBCL) living in lower SES neighborhoods had 34% and 24% higher mortality rate from all causes and lymphoma, respectively. The magnitude of mortality disparities was marked in younger patients (i.e. not eligible for Medicare) [27]. Coverage of trial-related expenses comes from a combination of the study sponsor, a patient’s insurance plan, and out-of-pocket expenses. Although insurance and the sponsor often pay for doctor visits and standard treatments, study drugs and additional tests are often not covered by insurance [28]. As some costs of a trial are reliant on insurance, patients with no insurance or with one that does not cover anticipated costs may limit their capacity to join [11]. total of 103 participants were enrolled in the study. Twenty-five were health care providers, 18 were clinic patients, and 60 were community participants; 24% lived in rural communities with populations less than 10,000. Patients and community participants ranged in age from 50 to 80 and were mostly female (81%),

Geographic location has become a more prominent variable in widening disparities after the introduction of CAR-T and bispecific antibodies. These therapies are often administered in specialized treatment centers which are not as widespread as community treatment centers, limiting access to individuals [29]. One study showed that patients from rural and Health Professional Shortage Area locations are less likely to receive immune effector cell therapies and demonstrate similar demographics to trial enrollment which indicates these populations enroll less in clinical trials [30]. Another study analyzed treatment sites that had CAR-T and bispecific antibody trials for diffuse large B-cell lymphoma across the US, and found that 20 states had no open trials, and that only a third of the Black population lived in a county with access to CAR-T or bispecific trial [31].

These disparities underscore the multifaceted nature of inequities within clinical trial enrollment, necessitating comprehensive strategies to address barriers and promote inclusivity in research participation. Table 1 summarizes these demographic and socioeconomic factors that contribute to the disparities seen in lymphoma clinical trials and treatment outcomes.

### Challenges in Increasing Diversity in Trials of Lymphoid Malignancies

Table 2 summarizes the recent research on the barriers to increasing diverse lymphoma clinical trial participation. Lymphoma and CAR-T cell clinical trials predominantly feature male, white participants residing in urban, more affluent areas and receiving care in academic medical centers [1, 17, 23]. Overrepresentation of these individuals threatens the external validity of the results and reduces the ability to generalize them to different racial, socioeconomic, geographic, and gender groups. These populations also have better overall survival rates, particularly in allogeneic hematopoietic cell transplantation trials.

**Table 2:** Barriers to CAR-T/bispecific trial enrollment

Reference	Date	Type of Study	Aim of Study	Barriers	Key Findings
Al Hadidi et al. [34]	2022	Retrospective cross-sectional	To examine the enrollment of Black participants in clinical trials that resulted in subsequent FDA approval of CAR-T products in hematologic malignant neoplasms	Race, ethnicity	• Significant disparities affect Black patients across approved CAR-T products. • Regulations are needed to ensure Black patient enrollment thresholds before FDA approval. • In the study supporting tisagenlecleucel for ALL, Black participants were categorized as “other,” with efficacy ranging from 2–5% in those who received the CAR-T product.
Jaggers et al. [18]	2021`	Retrospective cross-sectional	To analyze the restrictions that inclusion/exclusion criteria for CAR-T therapy in hematologic malignancies pose	Age, performance status, renal function	• CAR-T therapy trials vary in inclusion/exclusion criteria, often restricting upper age, performance status, and renal function. • These restrictions can limit patient access, highlighting the need for a standardized, evidence-based enrollment approach.
Bezerra et al. [48]	2023	Retrospective cohort	To evaluate the clinical trial eligibility barriers in patients with aggressive B-cell NHL with progression/relapse after CART19	CAR-T cell treatment failure	• 50% did not meet eligibility criteria for recent landmark or anti-CD20 bispecific antibody trials, primarily due to hematologic exclusion criteria. • Criteria may need adjustment to better reflect the needs and risks of this patient population.
Alqazaqi et al. [29]	2022	Retrospective cross-	Investigate current geographic distribution of CAR-T cell therapy and bispecific antibodies for multiple myeloma	Geographic, race	• Analysis of 162 clinical trials revealed that 35.9% of Black patients lived in counties with available trials. • In the 10 states with the highest proportion of Black residents (18.6–41.4%), 6 states had no or fewer than 3 openings for CAR-T or bispecific antibody studies.
Tabak et al. [30]	2024	Retrospective cross-sectional	Aims to compare the impact of rural and Health Professional Shortage Area (HPSA) backgrounds on immune effector cell clinical trial enrollment and standard of care (SOC) CAR-T treatments for NHL and MM.	Geographic, race	• Fewer patients from Health Professional Shortage Areas (HPSA) or rural regions received CAR-T therapy. • Rural HPSA patients were predominantly White, older, and lived further from trial sites. • No Black patients from rural areas were represented in these trials, highlighting a significant relationship between rurality and race.

### Strategies for Improving Diversity

**Fig. 2:**
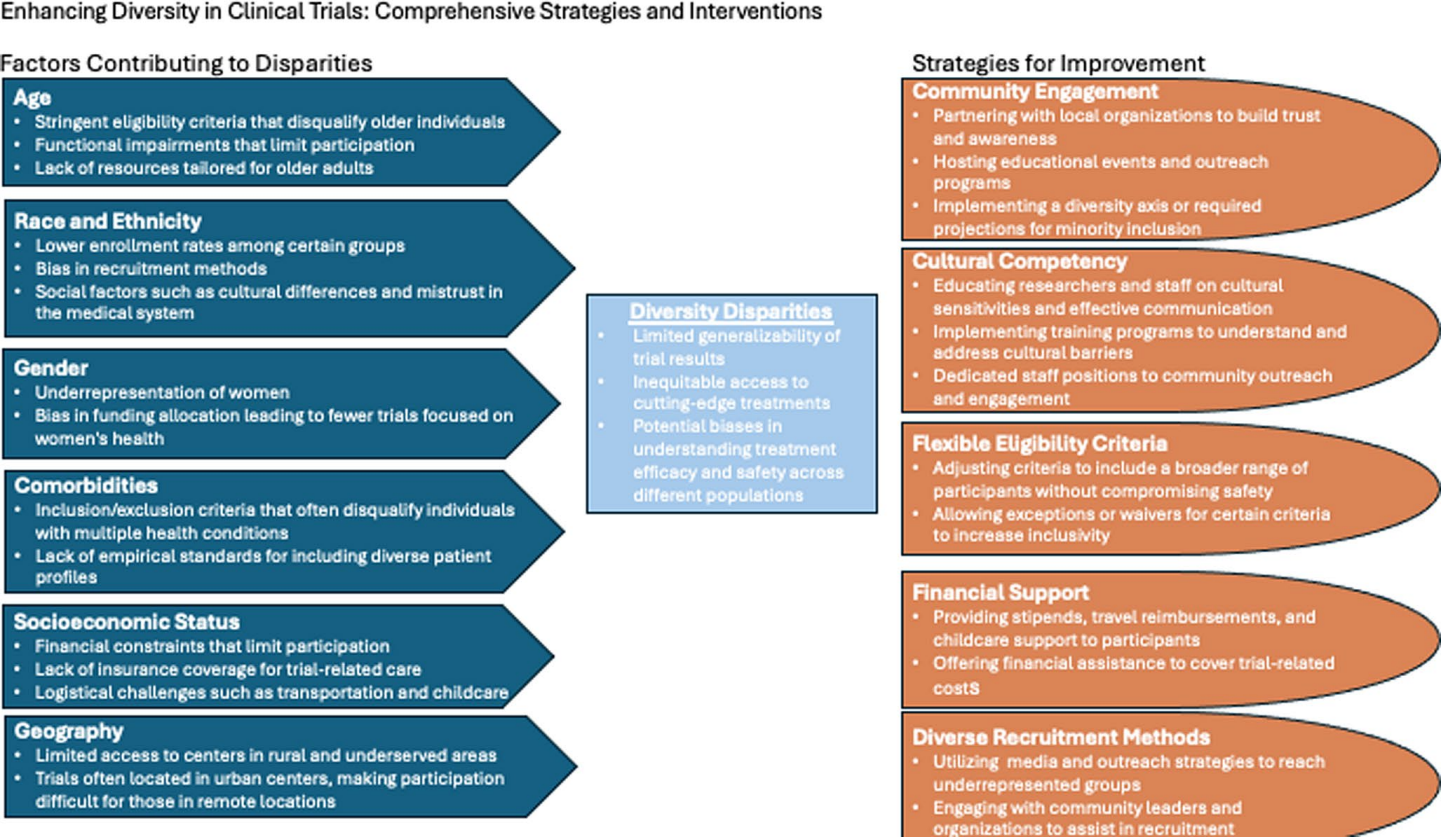
Factors contributing to disparities and strategies for improvement

To enhance diversity in clinical trial enrollment, multifaceted interventions at trial recruitment, enrollment, and continued participation are imperative to address various complex barriers encountered by underrepresented populations (Fig. 2). An example is the Minority Accrual Plan developed and implemented by the University of Texas health center [32].

### Improving Minority Recruitment

Enrollment to clinical trials starts with defining the eligibility of participants. Creating stringent inclusion and exclusion criteria such as restricting age, comorbidities, and disease spectrums excludes diverse participants and reduces the application of clinical trials to diverse individuals [33]. A potential strategy to ensure inclusion of diverse populations is creating a diversity axis which considers different factors and sets a predetermined number of participants per diversity category. Enrollment would remain open until all categories are saturated. The Minority Accrual Plan implements a similar strategy by requiring all new clinical trials to include projections for minority participation and strategies to overcome enrollment barriers and including a template and toolbox to guide these efforts [32]. Included resources are bilingual support, translated materials, and media outreach, all integrated into the trial approval process with the idea of increasing the quality and relevance of clinical trial data [32]. This approach presents several potential challenges, including the risk of bias in defining diversity, difficulty in recruiting patients for specific categories, and prolongation of the recruitment phase in clinical trials. For example, studies could enforce certain thresholds of demographics like black patients’ enrollment before granting FDA approval for products [34]. While standardizing diversity offers certain advantages, it also involves balancing the risk of exploiting minority groups for research purposes with the need to address diversity-related concerns.

Outreach programs targeting minority communities through various channels such as media outlets, churches, and health fairs can enhance the visibility of clinical trials [35]. Initiatives to educate individuals about the benefits of participation, dispel misconceptions/ firmly rooted cultural biases, and provide educational resources that empower patients to make informed decisions can further improve enrollment [36, 37]. Creating an informed consent process that is inclusive necessitates the use of simple language and culturally appropriate messages [37]. Any specific training curricula should also be developed to recognize and address race/ethnic cultural barriers [35]. Additionally, exploring minority participation fraction as a measure for institutional racial diversity can provide valuable insights into addressing disparities in correlative science participation [38].

Prospective studies are imperative for a comprehensive understanding and addressing of these disparities, with current literature predominantly emphasizing retrospective descriptive and qualitative studies [38]. Transparent reporting of demographic data in medical journals is crucial for advancing understanding and addressing disparities within the field [34]. From the staff level, efforts to increase the representation of under-represented clinicians, researchers and research staff are essential to promote diversity and inclusivity in medical research [39].

### Improving Access

As clinical trials necessitate numerous visits, it is important to consider the barriers related to the longitudinal nature of completing trials. Offering alternatives for trial communication and study visits, such as Telehealth, can improve continued accessibility, although technology-driven substitutes may not be as effective among underrepresented groups [37]. Transportation barriers are also cited as a major barrier for trial participation, thus including questions about travel concerns, monetary compensation for travel, or providing travel services could be considered [37]. Establishing partnerships between CAR-T centers and external hospitals can improve provider familiarity, facilitate patient referrals, and enhance overall access to CAR-T clinical trials [40]. Expanding CAR-T therapy to community hospitals and axillary buildings such as specialized cancer treatment centers offer the potential to reduce travel time and financial toxicity [23]. Periodic diversity surveys can be another way to assess progress towards improving access through continuous feedback from patients, investigators and community partners throughout a trial. Monitoring the diversity of patients enrolled in trials through recording sex, race and ethnicity could help to ensure that minority populations are represented.

### Accounting for Biologic Differences

From a biological standpoint, it is vital to recognize and account for racial differences in lab values to prevent the exclusion of potential participants based on normal variations related to race [35]. For instance, benign ethnic neutropenia is commonly observed in African Americans and can lead to exclusion from clinical trials due to Absolute Neutrophil Count (ANC) values below 1.0, despite this being a normal variant in this population [41]. This condition can result in unnecessary exclusion of African Americans from clinical trials and treatments that use ANC as a criterion, possible skewing research outcomes and treatment efficacy across different racial groups [41]. Increasing representation in biobanking is also essential to prevent the misdirection of drug development and promote pharmacogenomic advancements [42]. Pharmacogenomics investigating genetic variations, both heritable and somatic, influence drug responses and are underrepresented in minority populations [36]. For instance, Black patients with lymphoma were less likely to receive standard chemoimmunotherapy compared to White patients, despite its recommendation as the standard of care [34]. Promoting studies that no not require HLA matching in clinical trials can be one avenue to promote participation among diverse populations.

### Addressing the Patient-Clinician Relationship

As individuals may have any combination of barriers to enrollment, adopting a patient-centered approach through patient navigation models can support participants throughout the trial process [43]. Beyond physical obstacles, addressing emotional needs and promoting mental health resources are vital components of ensuring participant well-being. Building stronger patient-physician relationships, approaching patients with cultural sensitivity in mind, and ensuring self-explanatory information are essential for fostering trust and engagement [37].

### Contributing to Culture Change/ Community Outreach and Engagement

Policy changes are imperative to address disparities in clinical trial enrollment. Organizations, such as the American Society of Clinical Oncology (ASCO), have established committees and policies to tackle cancer care disparities and promote health equity. Community involvement is also crucial for enhancing access to trials among minority groups, necessitating collaboration with community hospitals, clinics, and agencies focused on serving underrepresented populations [35, 37]. Hospital systems can enhance community engagement by creating dedicated staff positions to identify local health needs and collaborate with community partners. Such Community Outreach and Engagement (COE) initiatives at various cancer centers can help improve access and outcomes for vulnerable populations. These roles can include participation in leadership boards and special projects, helping to stay connected with community needs and address enrollment disparities. Creating government incentives or funds for implementing trials in community hospitals can help apply these ideas. Utilizing telemedicine methods can broaden hospital eligibility for participation in research and Principal Investigator involvement. One example of COE is the Community Clinical Oncology Program funded by the NCI that aimed to increase community-based physicians’ familiarity with clinical trials to increase quality and accessibility of community-based care [44]. Another example is the Abramson Cancer Center’s COE program that creates culturally tailored marketing strategies in collaboration with faith-based organizations serving black communities with the goal of increasing enrollment of black patients into cancer clinical trials [44]. Implementing programs like these to advocate for minority populations and encourage their involvement in trials is integral for increasing access and improving diversity in clinical trials.

## Conclusion

Addressing the disparities in clinical trial enrollment requires a multifaceted approach from participant, physician, institutional, and system levels. From defining eligibility criteria to implementing recruitment strategies and considering logistical barriers, the challenges are diverse and complex. Strategies aimed at enhancing diversity must encompass educational initiatives, patient navigation models, policy changes, and community involvement. Recognizing and addressing the socioeconomic, biological, and institutional factors that contribute to enrollment disparities is crucial. Fostering trust, promoting inclusivity, and establishing partnerships between healthcare institutions are pivotal steps towards achieving equitable access to clinical trials for all individuals, irrespective of demographic backgrounds. Concerted efforts from all stakeholders are essential to bridge the gap in diversity and promote inclusivity in clinical research, thereby advancing medical advancements and addressing healthcare disparities effectively.

## Key References

- Jaggers, J.L., et al., *Characterizing inclusion and exclusion criteria in clinical trials for chimeric antigen receptor (CAR) T-cell therapy among adults with hematologic malignancies.* J Geriatr Oncol, 2021. **12**(2): p. 235–238.
- Alqazaqi, R., et al., *Geographic and Racial Disparities in Access to Chimeric Antigen Receptor-T Cells and Bispecific Antibodies Trials for Multiple Myeloma.* JAMA Netw Open, 2022. **5**(8): p. e2228877.
- Davis, T.C., et al., *A Qualitative Study Exploring Barriers and Facilitators of Enrolling Underrepresented Populations in Clinical Trials and Biobanking.* Front Cell Dev Biol, 2019. **7**: p. 74.
- Bezerra, E.D., et al., *Barriers to enrollment in clinical trials of patients with aggressive B-cell NHL that progressed after CAR T-cell therapy.* Blood Adv, 2023. **7**(8): p. 1572–1576.

## Data Availability

No datasets were generated or analysed during the current study.

## References

[1] Murthy VH, Krumholz HM, Gross CP. Participation in cancer clinical trials: race-, sex-, and age-based disparities. JAMA. 2004;291(22):2720–6. 15187053 10.1001/jama.291.22.2720

[2] H.R.6584–117th Congress (2021–2022): DEPICT Act. 2022 https://www.congress.gov/bill/117th-congress/house-bill/6584

[3] S.1701–118th Congress (2023–2024), NIH Clinical Trial Diversity Act of 2023; https://www.congress.gov/bill/118th-congress/senate-bill/1701/text

[4] Chen MS Jr., et al. Twenty years post-NIH revitalization act: enhancing minority participation in clinical trials (EMPaCT): laying the groundwork for improving minority clinical trial accrual: renewing the case for enhancing minority participation in cancer clinical trials. Cancer. 2014;120(Suppl):1091–6. 24643646 10.1002/cncr.28575 PMC3980490

[5] Locke FL, et al. Long-term safety and activity of axicabtagene ciloleucel in refractory large B-cell lymphoma (ZUMA-1): a single-arm, multicentre, phase 1–2 trial. Lancet Oncol. 2019;20(1):31–42. 30518502 10.1016/S1470-2045(18)30864-7 PMC6733402

[6] Schuster SJ, et al. Long-term clinical outcomes of tisagenlecleucel in patients with relapsed or refractory aggressive B-cell lymphomas (JULIET): a multicentre, open-label, single-arm, phase 2 study. Lancet Oncol. 2021;22(10):1403–15. 34516954 10.1016/S1470-2045(21)00375-2

[7] Abramson JS, et al. Lisocabtagene maraleucel for patients with relapsed or refractory large B-cell lymphomas (TRANSCEND NHL 001): a multicentre seamless design study. Lancet. 2020;396(10254):839–52. 32888407 10.1016/S0140-6736(20)31366-0

[8] Locke FL, et al. Axicabtagene Ciloleucel as Second-Line therapy for large B-Cell lymphoma. N Engl J Med. 2022;386(7):640–54. 34891224 10.1056/NEJMoa2116133

[9] Bishop MR, Purtill DM, Barba D, Santoro P, Hamad A, Kato N, Sureda K, Greil A, Thieblemont R, Morschhauser C, Janz F, Flinn M, Rabitsch I, Kwong W, Kersten YL, Minnema MJ, Holte MC, Chan H, Martinez-Lopez EHL, Müller J, Maziarz AMS, McGuirk RT, Bachy JP, Le Gouill E, Dreyling S, Harigae M, Bond H, Andreadis D, McSweeney C, Kharfan-Dabaja P, Newsome M, Degtyarev S, Awasthi E, Del Corral R, Andreola C, Masood G, Schuster A, Jäger SJ, Borchmann U, Westin P. J.R., Second-line tisagenlecleucel or standard care in aggressive B-cell lymphoma. The New England Journal of Medicine; 2021. p. 386. 10.1056/NEJMoa2116596 34904798

[10] Kamdar M, et al. Lisocabtagene maraleucel versus standard of care with salvage chemotherapy followed by autologous stem cell transplantation as second-line treatment in patients with relapsed or refractory large B-cell lymphoma (TRANSFORM): results from an interim analysis of an open-label, randomised, phase 3 trial. Lancet. 2022;399(10343):2294–308. 35717989 10.1016/S0140-6736(22)00662-6

[11] De Nardi M, et al. Medical spending of the US Elderly. Fisc Stud. 2016;37(3–4):717–47. 31404348 10.1111/j.1475-5890.2016.12106 PMC6680320

[12] Sedrak MS, et al. Older adult participation in cancer clinical trials: a systematic review of barriers and interventions. CA Cancer J Clin. 2021;71(1):78–92. 33002206 10.3322/caac.21638 PMC7854940

[13] Pittell H, et al. Racial and ethnic inequities in US Oncology Clinical Trial Participation from 2017 to 2022. JAMA Netw Open. 2023;6(7):e2322515. 37477920 10.1001/jamanetworkopen.2023.22515 PMC10362465

[14] Guadamuz JS et al. Multi- level factors underlying Racial/Ethnic inequities in clinical trial participation among patients with hematologic cancers: lessons for the development of Diversity Plans. Blood, 2023. 142.

[15] Horowitz MM. Transplantation and Cellular Therapy, racial and ethnic diversity on blood and marrow transplant clinical trials network (. BMT CTN) Trials– We Can Do Better; 2022. 10.1016/j.jtct.2024.10.014 PMC11735273 39489220

[16] Faruqi AJ, et al. The impact of race, ethnicity, and obesity on CAR T-cell therapy outcomes. Blood Adv. 2022;6(23):6040–50. 35939781 10.1182/bloodadvances.2022007676 PMC9700270

[17] Ahmed N, et al. Socioeconomic and Racial Disparity in Chimeric Antigen Receptor T cell Therapy Access. Transpl Cell Ther. 2022;28(7):358–64. 10.1016/j.jtct.2022.04.008 35429662

[18] Jaggers JL, et al. Characterizing inclusion and exclusion criteria in clinical trials for chimeric antigen receptor (CAR) T-cell therapy among adults with hematologic malignancies. J Geriatr Oncol. 2021;12(2):235–8. 32855108 10.1016/j.jgo.2020.08.004 PMC11892239

[19] Adams-Campbell LL, et al. Enrollment of African americans onto clinical treatment trials: study design barriers. J Clin Oncol. 2004;22(4):730–4. 14966098 10.1200/JCO.2004.03.160

[20] Cho Y, Shang S, Zhou W. Comorbidities were associated with cancer clinical trial discussion and participation: findings from the Health Information National Trends Survey-Surveillance, Epidemiology, and end results program (2021). J Clin Epidemiol. 2023;163:62–9. 37783400 10.1016/j.jclinepi.2023.09.016

[21] Flowers CR, et al. Disparities in the early adoption of chemoimmunotherapy for diffuse large B-cell lymphoma in the United States. Cancer Epidemiol Biomarkers Prev. 2012;21(9):1520–30. 22771484 10.1158/1055-9965.EPI-12-0466 PMC4155492

[22] Flowers CR, Nastoupil LJ. Socioeconomic disparities in lymphoma. Blood. 2014;123(23):3530–1. 24904097 10.1182/blood-2014-04-568766 PMC4047493

[23] Snyder S, et al. Access to Chimeric Antigen Receptor T cell therapy for diffuse large B cell lymphoma. Adv Ther. 2021;38(9):4659–74. 34302277 10.1007/s12325-021-01838-z PMC8408091

[24] Zafar SY, et al. The financial toxicity of cancer treatment: a pilot study assessing out-of-pocket expenses and the insured cancer patient’s experience. Oncologist. 2013;18(4):381–90. 23442307 10.1634/theoncologist.2012-0279 PMC3639525

[25] Snyder S, et al. Travel-Related Economic Burden of Chimeric Antigen Receptor T Cell Therapy Administration by Site of Care. Adv Ther. 2021;38(8):4541–55. 34279805 10.1007/s12325-021-01839-y PMC8342383

[26] Rachel Cusatis IT, Piehowski C, Akinola I, Crawford E, Craig J, Thiengmany A, Frank MJ, Miklos DB, Shah NN, Anita D, Souza, Jennifer M, Knight L, Muffly, Kathryn E, Flynn. Surbhi Sidana, worsening Financial Toxicity among patients receiving chimeric Antigen receptor t-Cell (CAR-T) therapy: a mixed methods longitudinal study. Blood. 2021;138:567.

[27] Tao L, et al. Socioeconomic disparities in mortality after diffuse large B-cell lymphoma in the modern treatment era. Blood. 2014;123(23):3553–62. 24705494 10.1182/blood-2013-07-517110 PMC4047495

[28] National Cancer Institute. Insurance coverage and clinical trials. National Cancer Institute, 2019.

[29] Alqazaqi R, et al. Geographic and racial disparities in Access to Chimeric Antigen Receptor-T Cells and bispecific antibodies trials for multiple myeloma. JAMA Netw Open. 2022;5(8):e2228877. 36018590 10.1001/jamanetworkopen.2022.28877 PMC9419017

[30] Tabak C. Chimeric Antigen Receptor T-Cell (CAR-T) Access Among Rural and Health Professional Shortage Area (HPSA) Populations in the Midwest. Transplantation and Cellular Therapy, 2024, ALIYA RASHID, SAM PEPPER, WILLIAM WESSON, AL-OLA ABDALLAH, FORAT LUTFI, MUHAMMED UMAIR MUSHTAQ, MARC HOFFMAN, SARAH BROMERT, ALLISON APPENFELLER. 30(2): p. S323.

[31] Shahzad M, et al. Geographic and Racial Disparities in Chimeric Antigen Receptor-T Cells and bispecific antibodies trials Access for diffuse large B-Cell lymphoma. Clin Lymphoma Myeloma Leuk. 2024;24(5):316–22. 38342727 10.1016/j.clml.2024.01.006

[32] Trevino M, et al. The development of a minority recruitment plan for Cancer clinical trials. J Community Med Health Educ. 2013;3(5):1000230. 25152846 10.4172/2161-0711.1000230 PMC4141778

[33] Brierley CK, et al. Low participation rates and disparities in participation in interventional clinical trials for myelodysplastic syndromes. Cancer. 2020;126(21):4735–43. 32767690 10.1002/cncr.33105

[34] Al Hadidi S, et al. Enrollment of black participants in pivotal clinical trials supporting US Food and Drug Administration Approval of Chimeric Antigen Receptor-T cell therapy for hematological malignant neoplasms. JAMA Netw Open. 2022;5(4):e228161. 35442451 10.1001/jamanetworkopen.2022.8161 PMC9021907

[35] Awidi M, Al Hadidi S. Participation of Black americans in Cancer clinical trials: current challenges and proposed solutions. JCO Oncol Pract. 2021;17(5):265–71. 33974816 10.1200/OP.21.00001 PMC8258017

[36] Dance KV, et al. Perceptions of clinical care and research among African-American patients with lymphoma. Leuk Lymphoma. 2021;62(8):1860–8. 33645400 10.1080/10428194.2021.1892092 PMC8760890

[37] Davis TC, et al. A qualitative study exploring barriers and facilitators of enrolling underrepresented populations in clinical trials and biobanking. Front Cell Dev Biol. 2019;7:74. 31114788 10.3389/fcell.2019.00074 PMC6502895

[38] Dressler LG et al. Participation in Cancer Pharmacogenomic studies: a study of 8456 patients registered to clinical trials in the Cancer and Leukemia Group B (Alliance). J Natl Cancer Inst, 2015. 107(10). 10.1093/jnci/djv188 PMC5758036 26160883

[39] Camidge DR, et al. Race and ethnicity representation in clinical trials: findings from a literature review of phase I oncology trials. Future Oncol. 2021;17(24):3271–80. 34047192 10.2217/fon-2020-1262

[40] Hall AG et al. Access to Chimeric Antigen Receptor T Cell Clinical Trials in underrepresented populations: a Multicenter Cohort Study of Pediatric and Young Adult Acute Lymphobastic Leukemia patients. Transpl Cell Ther, 2023. 29(6): p. 356 e1-356 e7. 10.1016/j.jtct.2023.03.022 PMC10247402 36966871

[41] Matthew Hsieh KC, Link B, Stroncek D, Wang E, Everhart J, Tisdale JF. Griffin Rodgers, Benign ethnic Neutropenia in individuals of African descent: incidence, Granulocyte mobilization, and Gene expression profiling. Blood, 2005. 106(11).

[42] Hantel A, et al. Inequities in Alliance Acute Leukemia Clinical Trial and Biobank participation: defining targets for intervention. J Clin Oncol. 2022;40(32):3709–18. 35696629 10.1200/JCO.22.00307 PMC9649272

[43] Brooks SE, et al. Increasing minority enrollment onto clinical trials: practical strategies and challenges emerge from the NRG Oncology Accrual Workshop. J Oncol Pract. 2015;11(6):486–90. 26464496 10.1200/JOP.2015.005934 PMC5706134

[44] Pohl SA, et al. Evolution of community outreach and engagement at National Cancer Institute-Designated Cancer Centers, an evolving journey. CA Cancer J Clin. 2024;74(4):383–96. 38703384 10.3322/caac.21841

[45] Christina Baggott MK, Holly SP, Pacenta L, Phillips CL. Inferior outcomes among black patients with childhood acute lymphoblastic leukemia following tisagenlecleucel. Transplantation & Cellular Therapy Meetings of ASTCT and CIBMTR; 2021.

[46] Kahn JM et al. An investigation of toxicities and survival in hispanic children and adolescents with ALL: results from the Dana-Farber Cancer Institute ALL Consortium protocol 05– 001. Pediatr Blood Cancer, 2018. 65(3). 10.1002/pbc.26871 PMC5766393 29090520

[47] Fiala MA, Wildes TM. Racial disparities in treatment use for multiple myeloma. Cancer. 2017;123(9):1590–6. 28085188 10.1002/cncr.30526 PMC5400674

[48] Bezerra ED, et al. Barriers to enrollment in clinical trials of patients with aggressive B-cell NHL that progressed after CAR T-cell therapy. Blood Adv. 2023;7(8):1572–6. 36219588 10.1182/bloodadvances.2022007868 PMC10139860

